# A Method for Optimizing the Dwell Time of Optical Components in Magnetorheological Finishing Based on Particle Swarm Optimization

**DOI:** 10.3390/mi15010018

**Published:** 2023-12-21

**Authors:** Bo Gao, Bin Fan, Jia Wang, Xiang Wu, Qiang Xin

**Affiliations:** 1 National Key Laboratory of Optical Field Manipulation Science and Technology, Chengdu 610209, China; gaobo20@mails.ucas.ac.cn (B.G.); wangjia@ioe.ac.cn (J.W.); wuxiang21@mails.ucas.ac.cn (X.W.); xinqiang@ioe.ac.cn (Q.X.); 2 Advanced Manufacturing Center of Optics, Chinese Academy of Sciences, Chengdu 610209, China; 3 Institute of Optics and Electronics, Chinese Academy of Sciences, Chengdu 610209, China; 4 University of Chinese Academy of Sciences, Beijing 100049, China

**Keywords:** optical manufacturing, magnetorheological finishing, dwell time, mid-spatial error

## Abstract

In this paper, a dwell time optimization method based on the particle swarm optimization algorithm is proposed according to the pulse iteration principle in order to achieve high-precision magnetorheological finishing of optical components. The dwell time optimization method explores the optimal solution in the solution space by comparing the accuracy value of the final surface with the set value. In this way, the dwell time optimization method was able to achieve global optimization of the overall dwell times and each dwell time point, ultimately realizing the high-precision processing of a surface. Through the simulation of two Φ156 mm asphaltic mirrors (1^#^ and 2^#^), the root-mean-square (RMS) and peak–valley (PV) values of 1^#^ converged from the initial values of 169.164 nm and 1161.69 nm to 24.79 nm and 911.53 nm. Similarly, the RMS and PV values of 2^#^ converged from the initial values of 187.27 nm and 1694.05 nm to 31.76 nm and 1045.61 nm. The simulation results showed that compared with the general pulse iteration method, the proposed algorithm could obtain a more accurate dwell time distribution of each point under the condition of almost the same processing time, subsequently acquiring a better convergence surface and reducing mid-spatial error. Finally, the accuracy of the optimization algorithm was verified through experiments. The experimental results demonstrated that the optimized algorithm could be used to perform high-precision surface machining. Overall, this optimization method provides a solution for dwell time calculation in the process of the magnetorheological finishing of optical components.

## 1. Introduction

Magnetorheological finishing (MRF), a new and representative advanced optical manufacturing method [1,2,3,4,5,6,7], has broad development prospects due to its advantages of strong stability in removal, high surface machining accuracy, and good surface and sub-surface quality [8,9,10]. It has been widely used in the processing of high-precision optical components [11]. Based on the Preston equation, MRF is a processing technique for correcting the surface error of optical components by presetting the trajectory, selecting the appropriate process parameters, and calculating the dwell time and corresponding residual error. Obtaining a highly precise dwell time determines the machining accuracy of the optical element.

Given that aspherical optics components can be used to correct phase difference, increase field of view, and improve image quality [12,13,14], and given the developments in their design technology, aspherical optics has been widely used [15]. However, due to the complexity of the surfaces of aspherical optical components and the fact that the requirements of modern optical systems regarding the surface accuracy, surface roughness, and subsurface damage of aspherical surfaces are more stringent, the corresponding manufacturing process is more difficult than that of spherical optical components [16,17,18]. Most previous studies employed an iterative method to calculate the dwell time of magnetorheological finishing to realize component machining [19] (pp. 25–29), but this method has problems related to low calculation accuracy and an obvious middle–high-frequency error [20] (pp. 131–138). As a parallel algorithm, particle swarm optimization (PSO) has high efficiency and relatively few parameters. More importantly [21] (pp. 12–15), it can be used to find the optimal solution in an iterative process [22,23,24]. Therefore, in order to achieve high-precision magnetorheological finishing of optical components, PSO was introduced into the dwell time calculation in this study, and an optimization algorithm based on PSO was then proposed to explore the optimal solution in the global optimization process. This method is a dwell time optimization algorithm designed to improve polishing quality. Based on the iteration method, the PSO algorithm realized the optimal selection of the dwell time point, thereby achieving the goal of high-precision surface machining. The feasibility of the scheme was verified via simulation processing on an aspherical surface, error compensation, and an experiment, and the ideal result was obtained.

## 2. Calculation Method of Dwell Time

Since the basic model for solving the dwell time is the convolution of the desired material removal function and the dwell time generated by the grinding head, the high-precision calculation of the dwell time and the post-processing algorithm are crucial for ultra-precision NC machining [25] (pp. 96–115).

The existing methods for determining dwell time include the Fourier transform method, the iteration method, and the linear equations method.

A. Fourier transform method

The Fourier transform method transforms a convolution operation into a product operation according to the equivalence between the convolution in the time domain and the product in the frequency domain. It requires the dwell points to be evenly divided. In addition, in order to ensure that the dwell time solution is not negative, the iterative parameters need to be adjusted several times. In recent years, the Fourier transform method has been widely applied to solve dwell time problems. Li et al. [26] determined dwell time using Fourier transform, which greatly reduced the number of convolution calculation processes.

B. Iteration method (PI)

Given the positive correlation between the dwell time of the optical element surface and the removal of surface error, the iterative method refers to obtaining the dwell time through several iterations based on linear time-invariant material removal theory. This method is widely used in computer-controlled machining due to its advantages of requiring a small number of calculations, having a fast calculation speed, and allowing for the acquisition of an ideal solution result. Nevertheless, the method also exhibits disadvantages, including low precision and an obvious error at middle and high frequencies. Zhou [27] (pp. 43–46) compared the characteristics of two iterative methods based on machining time and machining accuracy. Zhou [28] (pp. 37–38) added a relaxation factor in the process of correcting dwell time via the pulse iteration method, which allowed the rate of residual error convergence to be controlled. Wang et al. [29] proposed an adaptive iterative algorithm.

C. Linear equations method

The principle of the linear equations method is to discretize the surface error and dwell time according to the motion trajectory and then solve the corresponding linear equation. This method has limitations in its application due to its high computational complexity, morbid matrix state, and slow computational speed. Luo et al. [30] determined the dwell time in the computer-controlled optical surfacing (CCOS) of optical elements with large diameters using a non-negative least squares method based on a sparse matrix and researched the regularization of the method. Based on introducing the regularization weight factor into the dwell time matrix equation, Wu et al. [31] added extra removal amounts to expand the freedom of the dwell time solution. Deng et al. [32] used the Tikhonov regularization method to solve established linear equations about dwell time, and the regularization parameter was determined via the adaptive method without any prior knowledge. Zhou et al. [33] proposed the application of the truncated singular value decomposition (TSVD) method to solve a dwell time linear model, balancing the relationship between the incompatibility of the solution and the residual surface error. Shi et al. [34] solved a dwell time vector by using the non-negative least squares method. Dong et al. [35] solved a matrix equation through the Tikhonov regularization and least squares QR decomposition (LSQR) methods, and a constrained LSQR method was presented to increase the robustness of the damped factor. Cheng et al. [36] proposed a coefficient matrix method in which sparse matrix operations were used to construct and store linear equations, greatly reducing the memory required for computing dwell time.

In this paper, to solve the problems related to dwell time calculation encountered when using existing methods and achieve high-precision magnetorheological finishing of optical components, a particle swarm optimization (PSO) algorithm is introduced based on the pulse iteration method. Since the solution of the dwell time is a deconvolution process, for which there is no exact solution, and the dwell time is non-negative, exploring the optimal solution is therefore the main problem associated with the dwell time algorithm. The PSO method achieves the global optimization of the dwell time solution by judging the surface residual value; thus, each dwell time point can be optimally selected. Introducing a particle swarm into the dwell time calculation method offers the advantages of a fast calculation speed and high accuracy and can effectively improve the mid-spatial error.

## 3. Dwell Time Calculation by Particle Swarm Optimization Algorithm

### 3.1. Evaluation Criteria of Surface Error

In order to improve the surface quality of optical components after polishing, the root-mean-square (RMS) value was used to evaluate the surface error of the workpiece.

**Figure 1:**
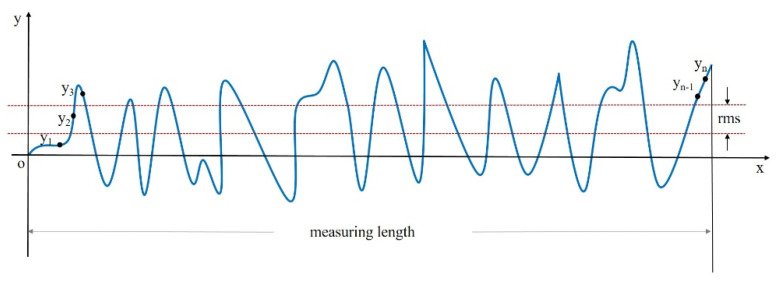
Schematic diagram of RMS calculation of surface error.

As shown in Figure 1, RMS is the root-mean-square deviation from the center line. This is a method for calculating an average value by squaring each value and then taking the square root of the average.

(1) $$\mathrm{RMS}={\left(\frac{y_{1}^{2}+y_{2}^{2}+y_{3}^{2}+\cdots +y_{n}^{2}}{n}\right)}^{1/2}$$

Here, *y_x_* is the height element along the outline, and *n* is the number of discrete elements. The RMS result is calculated as the standard deviation of the height (or depth) of the test surface relative to the reference at all data points in the dataset. The RMS result is the root-mean-square of the surface error or transmission error relative to the reference surface. The RMS result is an area-weighted statistic. In measuring the performance of optical components, RMS can be used to describe the optical performance of the component surface more accurately than PV statistics because it uses all the data in the associated calculations.

### 3.2. Establishing the Dwell Time Optimization Algorithm

The proposal of the particle swarm optimization (PSO) algorithm was inspired by the foraging behavior of birds [37,38]. Each particle in a particle swarm represents a possible solution to a problem. The core principle of the algorithm is to use the information-sharing behavior of each individual in the whole swarm to cause the motion of all particles to change iteratively; they then tend to move towards a specified solution space, finally obtaining the optimal solution [39] (pp. 29–39).

Supposing the surface error of the component to be machined is $Z\left(x,y\right)$ , the removal function generated by the polishing head in unit time on the surface of the optical element is $R\left(x,y\right)$ . In the process of iterative calculation, the dwell time obtained through a single iteration is $T\left(x,y\right)$ . Then, the residual difference between the removal amount calculated theoretically via the dwell time solution process and the expected removal amount based on the surface error, defined as $E\left(x,y\right)$ , can be calculated as follows:

(2) $$E\left(x,y\right)=Z\left(x,y\right)-R\left(x,y\right)*T\left(x,y\right)$$

where ∗ represents the convolution, the convolution of the removal function $R\left(x,y\right)$ with the dwell time $T\left(x,y\right)$ represents the material removed from the optical element within the resident time $T\left(x,y\right)$ , and the solution of the dwell time $T\left(x,y\right)$ is deconvolution.

If the removal function $R\left(x,y\right)$ is idealized as a removal pulse, the intensity of the removal pulse *I* is expressed as

(3) $$I=\iint_{a}^{b}R\left(x,y\right)dxdy$$

where *a* and *b* represent the upper and lower limits of the effective range interval in the removal function $R\left(x,y\right)$ , respectively.

The initial value of the dwell time $T_{0}\left(x,y\right)$ is set as

(4) $$T_{0}\left(x,y\right)=Z\left(x,y\right)/I$$

Then, the initial residual $E_{0}\left(x,y\right)$ is expressed as

(5) $$E_{0}\left(x,y\right)=Z\left(x,y\right)-R\left(x,y\right)*T_{0}\left(x,y\right)$$

According to the dwell time $T_{k}\left(x,y\right)$ , particle swarm optimization is introduced to obtain a new dwell time ${T_{k}}^{\prime }\left(x,y\right)$ . If the location of each dwell time point is treated as a particle, then the particle swarm $x\left(k\right)$ can be represented as follows:

(6) $$x\left(k\right)=\left(x_{1}\left(k\right),x_{2}\left(k\right),x_{3}\left(k\right)\cdots x_{i}\left(k\right)\right)$$

where *i* indicates the particle number, and *k* represents the *k* generation particle, i.e., the number of iterations of the particle. If $x_{i}\left(k\right)$ is substituted into the fitness function, the position of the *k* generation particle can be measured. The step size ( $v\left(k\right)$ ) of the *k* generation particle can be expressed as

(7) $$v\left(k\right)=\left(v_{1}\left(k\right),v_{2}\left(k\right),v_{3}\left(k\right)\ldots v_{i}\left(k\right)\right)$$

The historical optimal location of a single particle ( $p_{best}$ ), that is, the optimal dwell time at each dwell point, can be expressed as follows:

(8) $$p_{best}=\left(p_{1,bset},p_{2,bset},p_{3,bset}\cdots p_{1,bset}\right)$$

The RMS of each particle and the corresponding particle dwell time are recorded as the individual optimal solution of the particle, while the global optimal RMS and the corresponding particle dwell time are recorded as the global optimal solution of the particle swarm.

In each iteration, the particle velocity, position, individual optimal solution, and global optimal solution will be updated, and the formulae for particle velocity and position update are as follows:

(9) $$v_{i}\left(k+1\right)=w\cdot v_{i}\left(k\right)+c_{1}r_{1}\cdot \left(p_{i,best}-x_{i}\left(k\right)\right)+c_{2}r_{2}\cdot \left(p_{best}-x_{i}\left(k\right)\right)$$

(10) $$x_{i}\left(k+1\right)=x_{i}\left(k\right)+v_{i}\left(k+1\right)$$

where w is the inertia weight, and a larger w enables the particles to have greater inertia, thus enabling the particle swarm to explore a larger region in the entire solution space [40,41]; $r_{1}$ and $r_{2}$ are two independent random numbers with a uniform distribution of [0, 1], which introduces a certain level of uncertainty into the iterative process and is more conducive to finding the optimal solution; and $c_{1}$ and $c_{2}$ are the learning factors, which render the particles close to the optimal position in the population, that is, the optimal dwell time of each dwell time point. According to the particle-position-updating rules, namely, Equations (9) and (10), the direction and speed of particle updating are determined. After the particle position is updated and a new dwell time distribution is obtained, then the dwell time ${T_{k}}^{\prime }\left(x,y\right)$ after each iteration can be expressed as

(11) $${T_{k}}^{\prime }\left(x,y\right)=\sum p_{i,best}$$

The corresponding RMS is calculated, and if the new RMS is superior to the historical optimal solution for the particle, it is recorded as the new individual optimal solution. Similarly, the global optimal solution of the particle swarm is updated. After that, the search direction of the particle swarm can be re-determined according to the calculation result of the current iteration step.

The residual error ${E_{0}}^{\prime }\left(x,y\right)$ after processing a single dwell time is expressed as

(12) $${E_{0}}^{\prime }\left(x,y\right)=Z\left(x,y\right)-R\left(x,y\right)*{T_{0}}^{\prime }\left(x,y\right)$$

The corresponding iterative calculation is performed by taking the calculated residual error as the surface error $Z\left(x,y\right)$ of the component to be machined and repeating Equations (8)–(12). During optical component processing, the expected value of the component surface’s residual error should be set; when the residual error obtained via processing does not meet the expected value, iterative processing, i.e., dwell time superposition, is required:

(13) $${T^{\prime }}_{k}\left(x,y\right)={T^{\prime }}_{k-1}\left(x,y\right)+{T^{\prime }}_{k}(x,y)$$

When the expected value is reached, the iteration ends, and the residual error can be expressed as

(14) $${E_{k}}^{\prime }\left(x,y\right)=Z\left(x,y\right)-R\left(x,y\right)*{T_{k}}^{\prime }\left(x,y\right)$$

Then, the total dwell time distribution $T^{\prime }\left(x,y\right)$ after particle swarm optimization can be expressed as

(15) $$T^{\prime }\left(x,y\right)=\sum_{k=1}^{n}{T^{\prime }}_{k}\left(x,y\right)$$

where *n* represents the total number of iterations of the loop.

## 4. Simulation Analysis

**Figure 2:**
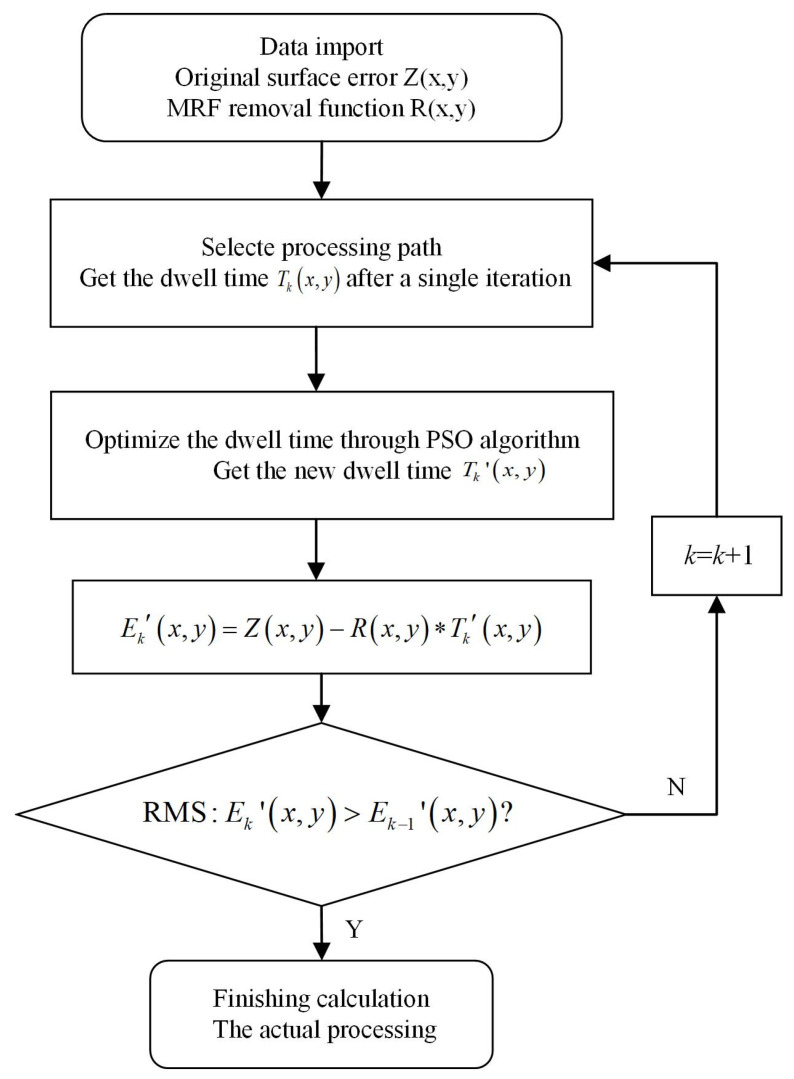
Process flow chart.

Off-axis aspherical mirrors 1^#^ and 2^#^, with a diameter of 156 mm, a curvature radius of −425.15 mm, an off-axis quantity of 121.23 mm, and a K coefficient of −1, were used as the components to be machined. The XY grating scanning path was used to carry out the simulation of magnetorheological finishing. The process flow chart is shown in Figure 2.

**Figure 3:**
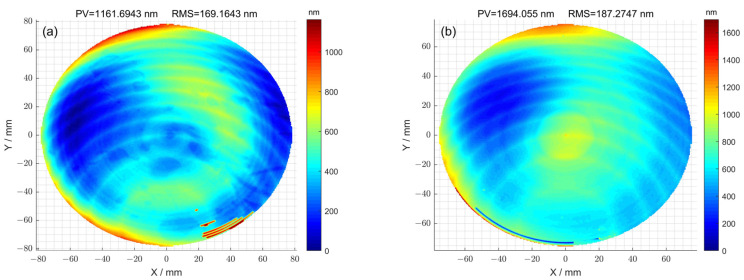
(**a**) Initial surface error of 1^#^ optical element; (**b**) initial surface error of 2^#^ optical element.

**Figure 4:**
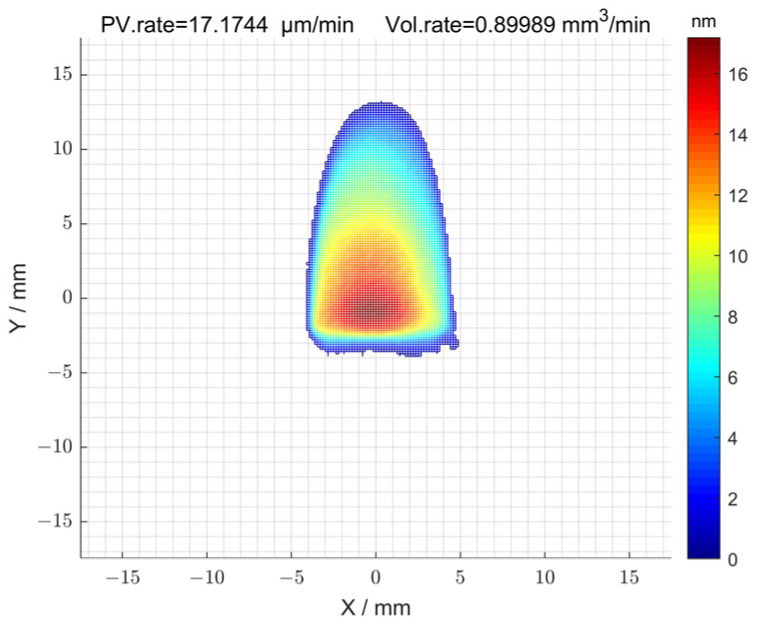
Removal function of MRF finishing.

The initial surface error distribution $Z\left(x,y\right)$ of 1^#^ is shown in Figure 3a, and its surface peak–valley (PV) and root-mean-square (RMS) values were 1161.69 nm and 169.16 nm, respectively. The initial surface error distribution $Z\left(x,y\right)$ of 2^#^ is shown in Figure 3b, and its surface peak–valley (PV) and root-mean-square (RMS) values were 1694.05 nm and 187.27 nm, respectively. The removal function used in the machining process is shown in Figure 4. The length and width of the removal function were 16 mm and 8 mm. In addition, the peak removal efficiency was 17.17 μm/min; the volume removal efficiency was 0.89 mm/min.

By substituting the surface error distribution $Z\left(x,y\right)$ and removal function $R\left(x,y\right)$ of the components to be processed into Equations (4) and (5), the initial dwell time $T_{0}\left(x,y\right)$ and corresponding surface residual error $E_{0}\left(x,y\right)$ of the pulse iteration method can be obtained. For the initial dwell time ${T_{0}}^{\prime }\left(x,y\right)$ , the dwell time distribution after particle swarm optimization can be obtained by optimizing according to Equations (9)–(11). Moreover, the residual error ${E_{0}}^{\prime }\left(x,y\right)$ after using the optimization method can be calculated using Equation (12).

When the pulse iteration method was not used for optimization, the calculated residual error $E_{0}\left(x,y\right)$ was taken as the surface error $Z\left(x,y\right)$ of the component to be machined and entered into the cycle for iterative calculation. When the particle swarm optimization algorithm was used, the calculated residual error ${E_{0}}^{\prime }\left(x,y\right)$ was taken as the surface error $Z\left(x,y\right)$ of the product to be machined and entered into the loop for iterative calculation.

**Figure 5:**
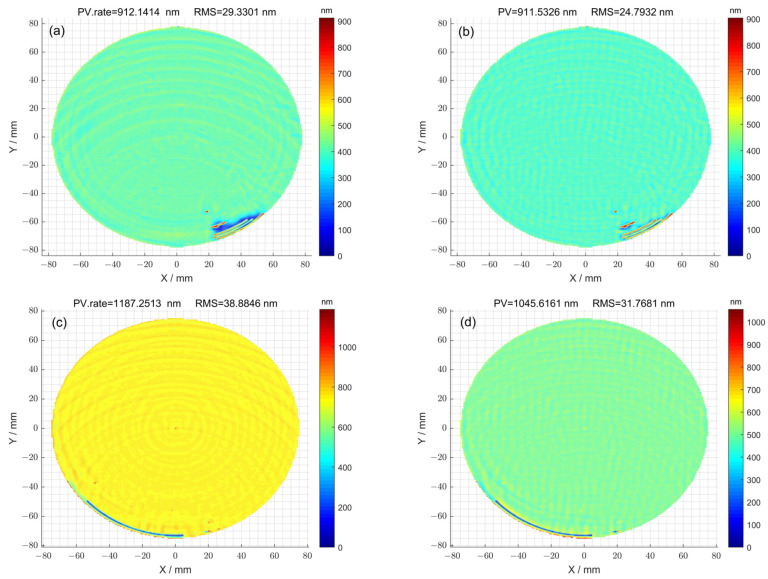
(**a**) The residual error of surface calculated using the pulse iteration method for 1^#^; (**b**) the residual error of surface calculated via the particle swarm optimization algorithm method for 1^#^; (**c**) the residual error of surface calculated using the pulse iteration method for 2^#^; (**d**) the residual error of surface calculated via the particle swarm optimization algorithm method for 2^#^.

In the case where the optimization method was not used for 1^#^, the residual error of the surface after seven iterations is shown in Figure 5a. The corresponding peak–valley (PV) value of the surface was 912.14 nm, and the root-mean-square (RMS) value was 29.33 nm. Using the optimization method, the residual error of the surface was obtained after thirteen iterations (Figure 5b). The corresponding peak–valley (PV) value of the surface was 911.53 nm, and the root-mean-square (RMS) value was 24.79 nm. In the case where the optimization method was not used for 2^#^, the residual error of the surface after eleven iterations is shown in Figure 5c. The corresponding peak–valley (PV) value of the surface was 1187.25 nm, and the root-mean-square (RMS) value was 38.88 nm. Using the optimization method, the residual error of the surface was obtained after fourteen iterations (Figure 5d). The corresponding peak–valley (PV) value of the surface was 1045.61 nm, and the root-mean-square (RMS) value was 31.76 nm. Compared with Figure 5a,c, the surface distributions corresponding to Figure 5b,d were smoother, and the peak–valley (PV) and root-mean-square (RMS) values were also smaller, indicating that the optimization method can improve the surface accuracy of components.

**Figure 6:**
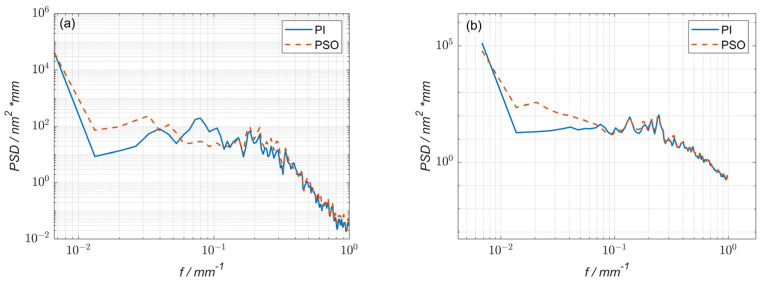
(**a**) The power spectral density (PSD) curve comparison of 1^#^ surface residual error; (**b**) the power spectral density (PSD) curve comparison of 2^#^ surface residual error.

By comparing the power spectral density (PSD) curves of the residual error of 1^#^ obtained using the particle swarm optimization and pulse iteration methods, it was found that, after using the optimization method, the PSD values decreased in the spatial frequency band of 0.05 mm^−1^ to 0.16 mm^−1^, indicating that the particle swarm optimization method can reduce the corresponding middle-and low-frequency surface errors during processing (Figure 6a). Although the spatial frequency was improved below 0.05 mm^−1^, the overall surface error peak–valley value (PV) and root-mean-square value (RMS) values were reduced; thus, this part could not be considered. By comparing the PSD curves of the residual error of 2^#^ obtained using the particle swarm optimization and pulse iteration methods, it was found that, after using the optimization method, the PSD values decreased in the spatial frequency band of 0.06 mm^−1^ to 0.16 mm^−1^, indicating that the particle swarm optimization method can reduce the corresponding middle-and low-frequency surface errors during processing (Figure 6b). Although the spatial frequency was improved to below 0.03 mm^−1^, the overall surface error PV and RMS values were reduced; thus, this part could not be considered.

**Figure 7:**
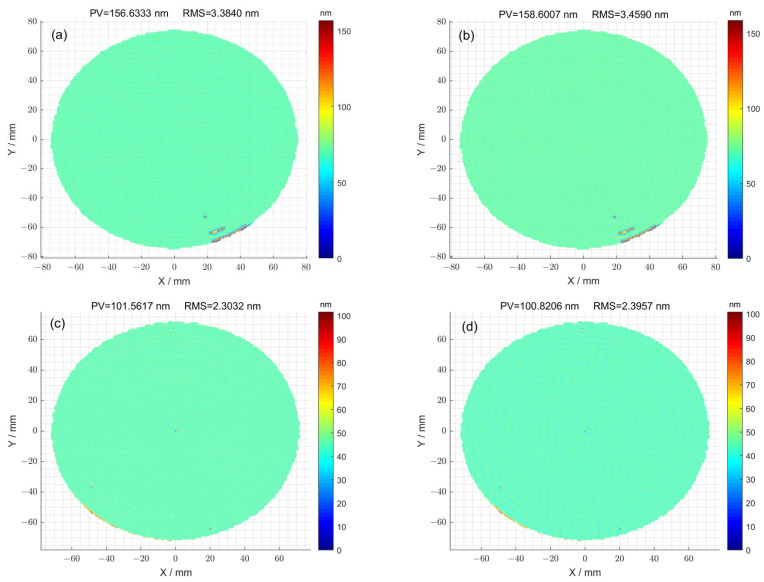
(**a**) The high-frequency error of surface calculated using pulse iteration method for 1^#^; (**b**) the high-frequency error of surface calculated using the particle swarm optimization algorithm method for 1^#^; (**c**) the high-frequency error of surface calculated using the pulse iteration method for 2^#^; (**d**) the high-frequency error of surface calculated via particle swarm optimization algorithm method for 2^#^.

In the case where the optimization method was not used for 1^#^, the high-frequency error of the surface is shown in Figure 7a. The corresponding peak–valley (PV) value of the surface was 156.63 nm, and the root-mean-square (RMS) value was 3.38 nm. The high-frequency error of the surface obtained using the optimization method is shown in Figure 7b. The corresponding peak–valley (PV) value of the surface was 158.60 nm, and the root-mean-square (RMS) value was 3.45 nm. For the case where the optimization method was not used for 2^#^, the high-frequency error of the surface is shown in Figure 7c. The corresponding peak–valley (PV) value of the surface was 101.56 nm, and the root-mean-square (RMS) value was 2.30 nm. The high-frequency error of the surface obtained using the optimization method is shown in Figure 7d. The corresponding peak–valley (PV) value of the surface was 100.82 nm, and the root-mean-square (RMS) value was 2.39 nm. Given that magnetorheological finishing is not sensitive to high-frequency information, the removal process is only applicable to low-frequency and mid-frequency errors. Therefore, the high-frequency errors obtained by filtering are similar regardless of whether they are processed using the pulse iteration method or the particle swarm optimization method.

**Figure 8:**
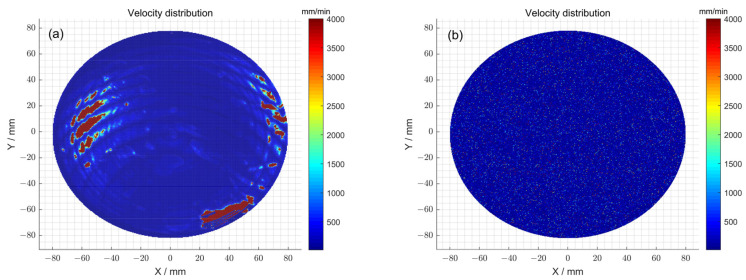

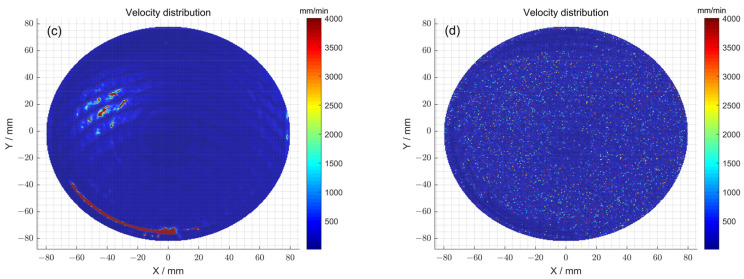
(**a**) The polishing wheel moving velocity distribution determined via the pulse iteration method for 1^#^; (**b**) the polishing wheel moving velocity distribution determined via particle swarm optimization algorithm method for 1^#^; (**c**) the polishing wheel moving velocity distribution determined via the pulse iteration method for 2^#^; (**d**) the polishing wheel moving velocity distribution determined via the particle swarm optimization algorithm method for 2^#^.

Furthermore, the feed velocity distributions of the polishing wheel for 1^#^ obtained with and without the particle swarm optimization method were compared (Figure 8a,b). Specifically, when the particle swarm optimization method was not used, the total dwell time distribution $T\left(x,y\right)$ obtained after the seventh iteration was satisfied: $T\left(x,y\right)=\sum_{k=0}^{6}T_{k}\left(x,y\right)$ . The feed speed corresponds to a peak–valley (PV) value of 4000 mm/min and a root-mean-square (RMS) value of 507.20 mm/min. In comparison, by using the particle swarm optimization method, the total dwell time distribution $T^{\prime }\left(x,y\right)$ obtained after the thirteenth iteration was satisfied: $T^{\prime }\left(x,y\right)=\sum_{k=0}^{12}{T_{k}}^{\prime }\left(x,y\right)$ . The feed speed corresponds to a peak–valley (PV) value of 4000 mm/min and a root-mean-square (RMS) value of 205.85 mm/min. The feed velocity distributions of the polishing wheel for 2^#^ obtained with and without the particle swarm optimization method were also compared (Figure 8c,d). Specifically, when the particle swarm optimization method was not used, the total dwell time distribution $T\left(x,y\right)$ obtained after the eleventh iteration was satisfied: $T\left(x,y\right)=\sum_{k=0}^{10}T_{k}\left(x,y\right)$ . The feed speed corresponds to a peak–valley (PV) value of 4000 mm/min and a root-mean-square (RMS) value of 350.41 mm/min. In comparison, by using the particle swarm optimization method, the total dwell time distribution $T^{\prime }\left(x,y\right)$ obtained after the fourteenth iteration was satisfied: $T^{\prime }\left(x,y\right)=\sum_{k=0}^{13}{T_{k}}^{\prime }\left(x,y\right)$ . The feed speed corresponds to a peak–valley (PV) value of 4000 mm/min and a root-mean-square (RMS) value of 178.44 mm/min. The above observations indicate that after using the optimization method, although the peak–valley (PV) value of the polishing wheel’s feed speed obtained via solving did not change, the root-mean-square (RMS) value of the obtained polishing wheel feed speed decreased, and the speed distribution was gentler. Therefore, it can be concluded that the optimization method can render the polishing wheel’s feed speed more uniform, reduce instantaneous acceleration and deceleration movement, and ensure the stability of the machine tool. Thus, the particle swam optimization method reduced the introduction of mid-spatial errors and ensured the high precision of the processing of the surface.

In the process of solving the dwell time, it is necessary to set the expected value of the surface residual error. When the residual error does not reach the expected value, the dwell time needs to be superimposed *k* + 1 times until it reaches the expected value or the surface no longer converges, and then the number of iterations of the dwell time and the total dwell time can be obtained. After using the optimization method, both the dwell time of each point and the residual error changed, resulting in the iterations of the dwell time being different from those obtained without the optimization method.

**Figure 9:**
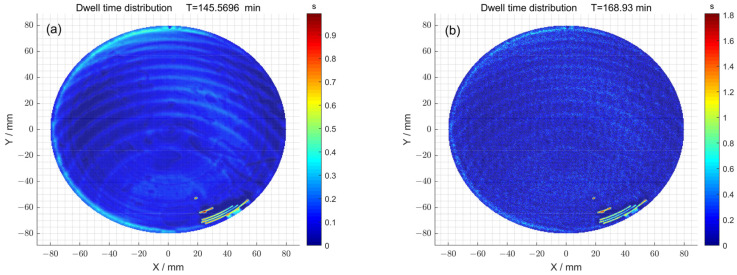

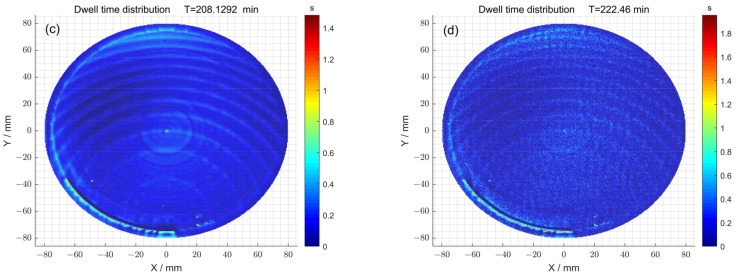
(**a**) The dwell time distribution obtained via the pulse iteration method of 1^#^; (**b**) the dwell time distribution obtained via the particle swarm optimization algorithm method of 1^#^; (**c**) the dwell time distribution obtained via the pulse iteration method of 2^#^; (**d**) the dwell time distribution obtained via the particle swarm optimization algorithm method of 2^#^.

Finally, the dwell time distributions of 1^#^ obtained with and without the particle swarm optimization method were compared (Figure 9a,b). When the particle swarm optimization method was not used, the total dwell time required was 145.56 min. Meanwhile, when particle swarm optimization was used, the total dwell time required was 168.93 min. The dwell time distributions of 2^#^ obtained with and without the particle swarm optimization method were also compared (Figure 9c,d). When the particle swarm optimization method was not used, the total dwell time required was 208.12 min. Meanwhile, when particle swarm optimization was used, the total dwell time required was 222.46 min. Consequently, in the case where there was no significant difference in dwell time, the surface accuracy of the component was improved by using particle swarm optimization.

In the area of the optimization of dwell time, the particle swarm optimization algorithm is a global optimization process. Therefore, to obtain a better surface, the optimization of each dwell point should be achieved during the calculation process, thereby achieving better control of the surface. However, this requires more iterations in the dwell time, thus increasing the processing time.

The simulation results for 1^#^ and 2^#^ are shown in Table 1. It can also be concluded that with almost the same dwell time, particle swarm optimization can increase the uniformity of the feed speed of the polishing wheel in the machining process, reduce instantaneous acceleration and deceleration movement, and ensure the stability of the machine tool during machining. Therefore, particle swarm optimization reduced the introduction of mid-spatial error, subsequently improving the surface accuracy of the components after processing.

## 5. Experimental Verification

**Figure 10:**
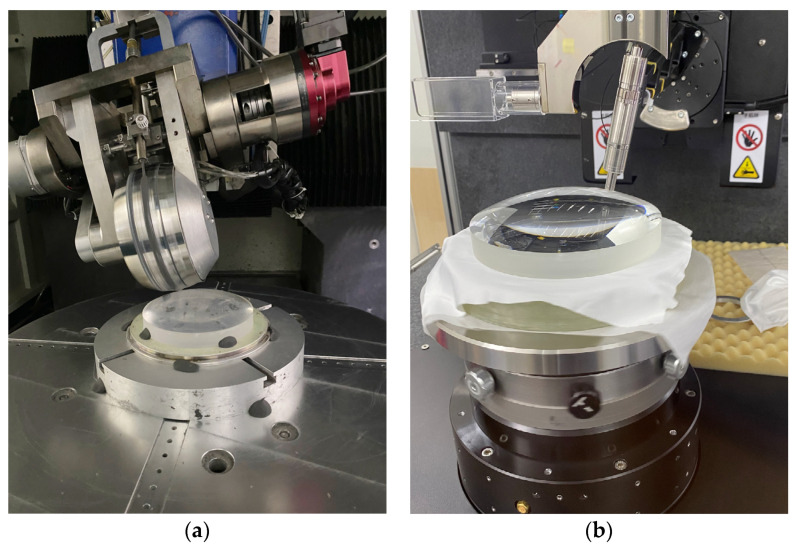
(**a**) Magnetorheological finishing; (**b**) surface detection.

In order to verify the performance of the optimization algorithm, an experimental test was conducted with an aspheric mirror. The processing and testing platforms are shown in Figure 10a,b, respectively. The processing equipment for the components was employed using a KDUPF-650 magnetorheological finishing machine (National University of Defense Technology, Changsha, China.). The precise detection of the surface was performed using LuphoScan-600HD (TAYLOR HOBSON, Leicester, UK), a high-speed non-contact 3D optical surface measurement system, and the scanning process was carried out using multi-wavelength interference (MWLI) technology. The components to be processed were aspherical K9 mirrors with a diameter of 148 mm, a curvature radius of 162.75 mm, and a K coefficient of −0.5319. An XY grating scanning path was used to carry out magnetorheological finishing. The magnetorheological slurry was mainly composed of a bi-phase base fluid, carbonyl iron particles (CIPs), additives, a pH regulator, and abrasive particles.

**Figure 11:**
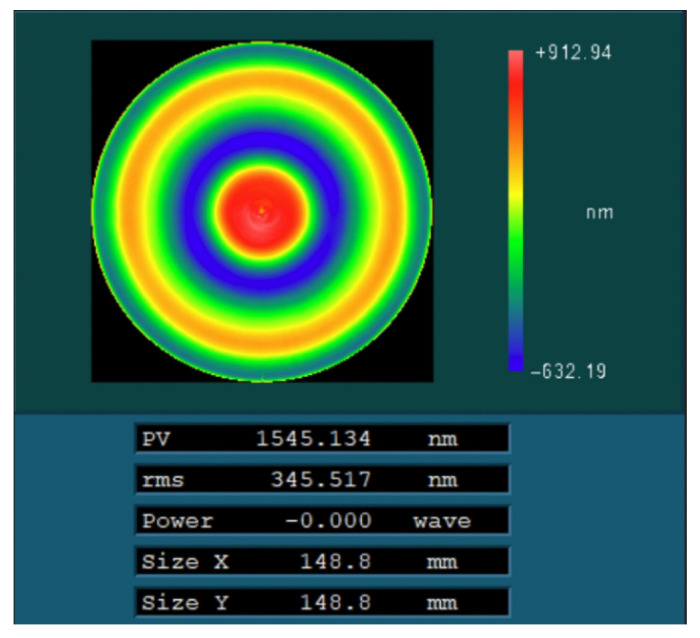
Initial surface error of optical element.

The initial surface error distribution of the component after the initial detection is shown in Figure 11. The peak-and-valley (PV) value of the surface was 1545.13 nm, and the root-mean-square (RMS) value was 345.51 nm. The removal function used during processing is also shown in Figure 4.

Before processing, the two processing methods were also compared via simulation. Because the initial surface distribution of the optical element used for processing was different from the simulated surface in Section 3, based on the characteristics of magnetorheological finishing, only a small amount of surface error was removed in the machining process.

**Figure 12:**
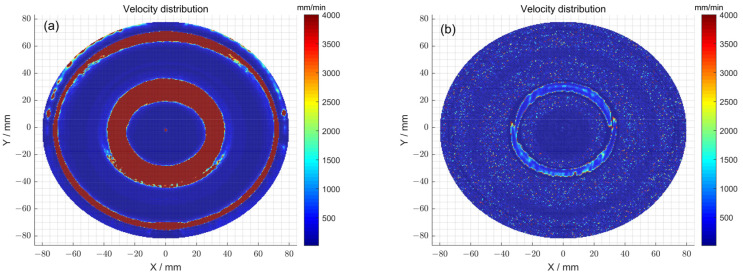
(**a**) The polishing wheel moving velocity distribution obtained using the pulse iteration method; (**b**) the polishing wheel moving velocity distribution obtained using the particle swarm optimization algorithm method.

First, the feed velocity distributions of the polishing wheel obtained with and without the particle swarm optimization method were compared through simulation (Figure 12a,b). Specifically, when the particle swarm optimization method was not used, the total dwell time distribution $T\left(x,y\right)$ obtained after the seventh iteration was satisfied: $T\left(x,y\right)=\sum_{k=0}^{6}T_{k}\left(x,y\right)$ . The feed speed corresponds to a peak–valley (PV) value of 4000 mm/min and a root-mean-square (RMS) value of 439.89 mm/min. In comparison, by using the particle swarm optimization method, the total dwell time distribution $T^{\prime }\left(x,y\right)$ obtained after the eleventh iteration was satisfied: $T^{\prime }\left(x,y\right)=\sum_{k=0}^{10}{T_{k}}^{\prime }\left(x,y\right)$ . The feed speed corresponds to a peak–valley (PV) value of 4000 mm/min and a root-mean-square (RMS) value of 124.74 mm/min. The above observations indicate that after using the optimization method, although the peak–valley (PV) value of the polishing wheel’s feed speed obtained via solving did not change, the root-mean-square (RMS) value of the obtained polishing wheel feed speed decreased, and the speed distribution was gentler. Therefore, the optimization method can increase the uniformity of the polishing wheel’s feed speed, reduce instantaneous acceleration and deceleration movement, and ensure the stability of the machine tool. Thus, particle swarm optimization reduced the introduction of mid-spatial error and ensured the high precision of the processing of the surface.

**Figure 13:**
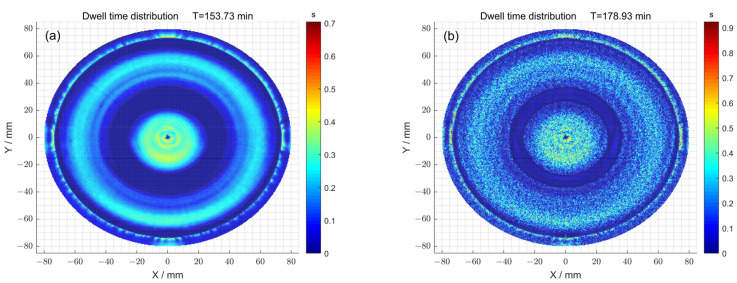
(**a**) The dwell time distribution obtained using the pulse iteration method; (**b**) the dwell time distribution obtained using the particle swarm optimization algorithm method.

Secondly, the dwell time distributions obtained with and without particle swarm optimization were compared (Figure 13a,b). When the particle swarm optimization method was not used, the total dwell time required was 153.73 min, while a dwell time distribution of 178.93 min was obtained using particle swarm optimization.

**Figure 14:**
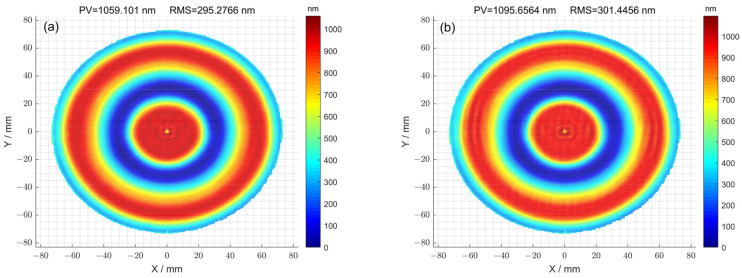
(**a**) The residual error of the surface calculated using the pulse iteration method; (**b**) the residual error of the surface calculated using the pulse iteration method.

Then, after processing using the two methods, the surfaces were compared through simulation. For the case where the optimization method was not used, the residual error of the surface after seven iterations is shown in Figure 14a. The corresponding peak–valley (PV) value of the surface was 1095.65 nm, and the root-mean-square (RMS) value was 301.44 nm. For the case where the optimization method was used, the residual error of the surface after eleven iterations is shown in Figure 14b. The corresponding peak–valley (PV) value of the surface was 1059.10 nm, and the root-mean-square (RMS) value was 295.27 nm. Compared with Figure 14a, the surface distribution corresponding to Figure 14b was smoother, and the peak–valley (PV) and root-mean-square (RMS) values were also smaller, demonstrating that the optimization method can improve the surface accuracy of components.

**Figure 15:**
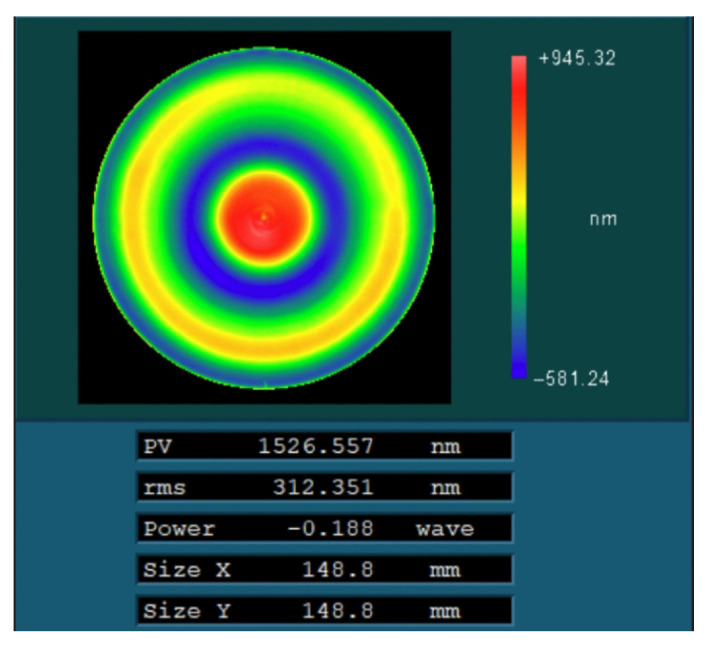
The residual error of the surface after actual machining.

Finally, the particle swarm optimization algorithm was used to process the optical components. The polishing wheel velocity distribution (Figure 12b) and dwell time distribution (Figure 13b) calculated by the above particle swarm optimization algorithm were tested to verify the process. The surface after processing is shown in Figure 15, and the corresponding peak–valley (PV) and root mean square (RMS) values were 1526.55 nm and 312.35 nm, respectively.

**Figure 16:**
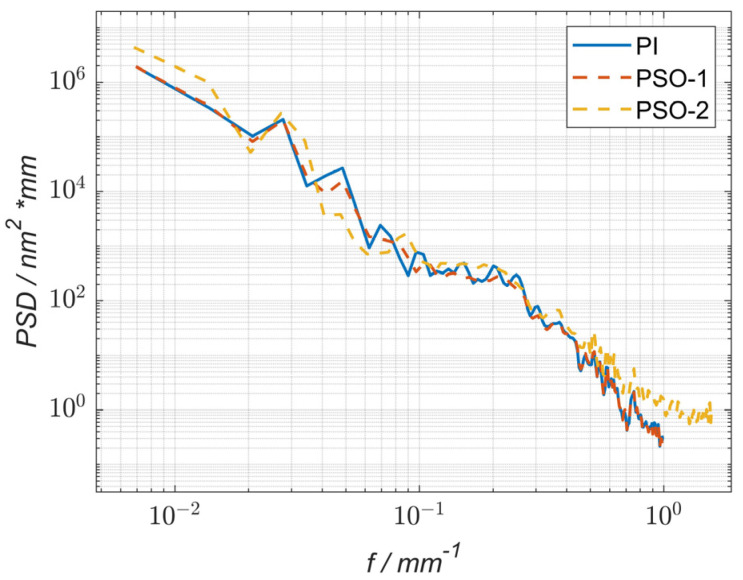
Power spectral density (PSD) curve comparison of the surface residual errors.

The residual power spectral density curves of the simulation process using the particle swarm optimization (PSO-1) method and the pulse iteration method (PI) were compared with the residual power spectral density curve of the actual process using the particle swarm optimization method (PSO-2), as shown in Figure 16. The results show that after using the optimization method (PSO-1), the PSD values in the frequency range of 0.03 mm^−1^ to 0.37 mm^−1^ were reduced compared to those obtained using the pulse iteration method (PI), indicating that the particle swarm optimization method can reduce the corresponding middle- and low-frequency surface errors in the machining process. Under the condition of a similar convergence rate and dwell time, lower PV and RMS values could be obtained after optimization. After the machining experiment, compared with the PSO-2 and PI curves, the PSD value of the spatial frequency in the band from 0.035 mm^−1^ to 0.16 mm^−1^ decreased, indicating that the optimization algorithm effectively reduced the middle- and low-frequency bands in the actual machining process. In addition, there was no significant difference in the PSD values between the values of the PSO-2 and PSO-1 curves in the spatial frequency band of 0.16 mm^−1^ to 0.37 mm^−1^, and this finding was attributed to an error in the actual machining process.

Table 2 shows the results obtained in the experimental process. The results indicate that the values obtained in the actual optimization process did not reach the theoretical value due to the existence of errors. Nonetheless, after using particle swarm optimization, the feed speed of the polishing wheel in the machining process was more uniform. Moreover, particle swarm optimization reduced instantaneous acceleration and deceleration movement and ensured the stability of the machine tool during machining, thus reducing the introduction of mid-spatial errors and improving the surface accuracy of the components after processing.

## 6. Conclusions and Implications

In the process of magnetorheological grid track machining, the different dwell times of each dwell point will affect the final result of polishing. In this paper, in order to achieve an accurate solution for the dwell time in the process of the magnetorheological finishing of optical components, the optimal dwell time distribution of each dwell point was explored. Furthermore, an optimized pulse iterative dwell time calculation algorithm based on particle swarm optimization was proposed to achieve high-precision machining of optical components. The optimal surface error distribution after magnetorheological finishing was obtained by introducing the particle swarm optimization algorithm into the model, setting up the objective function, and searching for the optimal solution in the solution space. After optimization, both RMS and PV were reduced, and the feed speed of the polishing wheel during processing was more uniform, which, in turn, reduced instantaneous acceleration and deceleration motion, ensured the stability of the machine tool during processing, and reduced the number of mid-spatial errors introduced. More importantly, this optimization algorithm is not limited to aspherical surface types and can be applied to the processing of any mirrored surface. Therefore, this method has important practical value for achieving high-precision magnetorheological finishing of optical components.

## Figures and Tables

**Table 1:** Simulation results of 1^#^ and 2^#^.

Index	Pulse Iteration Method of 1^#^	Particle Swarm Optimization of 1^#^	Pulse Iteration Method of 2^#^	Particle Swarm Optimization of 2^#^
Initial surface error PV/nm	1161.69	1161.69	1694.05	1694.05
Initial surface error RMS/nm	169.16	169.16	187.27	187.27
Surface error after machining PV/nm	912.14	911.53	1187.25	1045.61
Surface error after machining RMS/nm	29.33	24.79	38.88	31.76
Polishing wheel feed speed PV/mm/min	4000	4000	4000	4000
Polishing wheel feed speed RMS/mm/min	507.20	205.85	350.41	178.44
Total dwell time of machining/min	145.56	168.93	208.12	222.46

**Table 2:** Experimental results.

Index	Pulse Iteration Method	Particle Swarm Optimization
Initial surface error PV/nm	1545.13	1545.13
Initial surface error RMS/nm	345.51	345.51
Surface error after simulation machining PV/nm	1095.65	1095.10
Surface error after simulation machining RMS/nm	301.44	295.27
Polishing wheel feed speed PV/mm/min	4000	4000
Polishing wheel feed speed RMS/mm/min	439.88	124.74
Total dwell time of machining/min	153.73	178.93
Surface error after actual machining PV/nm	-	1526.55
Surface error after actual machining RMS/nm	-	312.35

## Data Availability

Data are contained within the article.
